# Atmospheric CO_2_ captured by biogenic polyamines is transferred as a possible substrate to Rubisco for the carboxylation reaction

**DOI:** 10.1038/s41598-018-35641-8

**Published:** 2018-12-07

**Authors:** Ko Yasumoto, Tsuyoshi Sakata, Jun Yasumoto, Mina Yasumoto-Hirose, Shun-ichi Sato, Kanami Mori-Yasumoto, Mitsuru Jimbo, Takenori Kusumi, Shugo Watabe

**Affiliations:** 1 Kitasato University School of Marine Biosciences, 1-15-1 Kitasato, Minami, Sagamihara, Kanagawa 252-0373 Japan; 2 Biological Laboratory, Center for Natural Sciences, College of Liberal Arts and Sciences, Kitasato University, 1-15-1 Kitasato, Minami, Sagamihara, Kanagawa 252-0373 Japan; 3 Department of Regional Agricultural Engineering, Faculty of Agriculture, University of the Ryukyus, 1 Senbaru, Nishihara, Nakagusuku, Okinawa, 903-0213 Japan; 4 Tropical Technology Plus Co., 12-75 Suzaki, Uruma, Okinawa, 904-2234 Japan; 5 Kagawa School of Pharmaceutical Sciences, Tokushima Bunri University, 1314-1 Shido, Sanuki, Kagawa, 769-2193 Japan; 6 Graduate School of Science and Engineering, Tokyo Institute of Technology, 2-12-1 Oookayama, Meguro-ku, Tokyo, 152-8551 Japan

## Abstract

Biogenic polyamines are involved in a wide range of plant cellular processes, including cell division, morphogenesis and stress responses. However, the exact roles of biogenic polyamines are not well understood. We recently reported that biogenic polyamines that have multiple amino groups can react with CO_2_ and accelerate calcium carbonate formation in seawater. The ability of biogenic polyamines to capture atmospheric CO_2_ prompted us to examine their roles in photosynthesis. Here, we demonstrated that atmospheric CO_2_ captured by biogenic polyamines is a candidate substrate for the carboxylation reaction of ribulose 1,5-bisphosphate carboxylase/oxygenase (Rubisco), which is an enzyme involved in the first major step of carbon fixation during photosynthesis, and that biogenic polyamines can accelerate the carboxylation reaction of this enzyme because of their specific affinity for CO_2_. Moreover, the results of our nuclear magnetic resonance (NMR) analysis showed that putrescine, which is the most common biogenic polyamine, reacts with atmospheric CO_2_ and promotes the formation of carbamate derivatives and bicarbonate in aqueous environments. A sufficient amount of CO_2_ is well known to be produced by carbonic anhydrase from bicarbonate *in vivo*. The present study indicates that CO_2_ would be also produced by the equilibrium reaction from carbonate produced by biogenic polyamines and would be used as a substrate of Rubisco, too. Our results may suggest a new photosynthetic research strategy that involves CO_2_-concentrating mechanisms and also possibly constitutes a potential tool for reducing atmospheric CO_2_ levels and, consequently, global warming.

## Introduction

The carboxylation reaction that fixes atmospheric CO_2_ into organic compounds during photosynthesis is the first reaction of organic synthesis. This reaction, which is catalyzed by ribulose 1,5-bisphosphate carboxylase/oxygenase (Rubisco), combines CO_2_ with ribulose 1,5-bisphosphate (RuBP) and is the main rate-limiting reaction of photosynthesis^1–4^. Although Rubisco is a large enzyme that has a molecular mass of approximately 550 kDa, the maximum reaction rate at 25 °C is only 15 to 30 mol CO_2_ mol^−1^ Rubisco s^−1^ ^4^. Furthermore, the affinity of Rubisco for CO_2_ is low: the Michaelis constant (K_m_) for CO_2_ at 25 °C is comparable to that of CO_2_ in water equilibrated with the atmosphere^3,4^. Therefore, plants require large amounts of Rubisco, and approximately half of leaf protein comprises this enzyme^4^.

The CO_2_ used for photosynthesis in terrestrial plants diffuses from the atmosphere into the leaves through the stomata. This CO_2_ then dissolves in the liquid phase of the mesophyll cell wall surface and reaches the Rubisco in the stroma of the chloroplast via the cell membrane, cytoplasm, and chloroplast envelopes^5^. This diffusion process substantially decreases the CO_2_ concentration. For example, in the leaf intercellular spaces, the concentration of CO_2_ is reduced to 60–85% that of the atmosphere, and in the stroma, the CO_2_ concentration is further reduced by 50–80%^5–9^. Thus, the decrease in CO_2_ reduces the rate of photosynthesis by approximately 2/3–1/2, and approximately half of this reduction is due to the decrease in the diffusion process from the intercellular spaces to the stroma^10,11^. In addition to simple diffusion mentioned above, diffusion-promoting proteins such as carbonic anhydrase and aquaporins facilitate CO_2_ diffusion within mesophyll cells^12–17^. These diffusion-promoting proteins occur in the cell membrane of mesophyll cells, and aquaporin amount, which can be manipulated by altering expression levels, affects mesophyll conductance (*g*_*m*_) in plants^18^. It is therefore necessary to accumulate more data on the characteristics and roles of the promoting mechanisms of CO_2_ diffusion in leaves^4,19,20^.

We recently reported that biogenic polyamines can capture atmospheric CO_2_ and accelerate bicarbonate/carbonate formation in aqueous solutions; these findings consequently led to the formation of extracellular bacterial CaCO_3_ ^21^. Polyamines are generally considered low-molecular-weight compounds that have multiple amino groups, are present at high concentrations in the cells of all organisms and are essential for both cell differentiation and proliferation^22–25^. In plants, the intracellular concentrations of polyamines are a few hundred µM to mM order^26^. It has been reported that polyamines are localized in the vacuoles, mitochondria and chloroplasts^26^. Moreover, chloroplasts contain a large amount of polyamines with high activities of the main polyamine biosynthetic enzymes ornithine decarboxylase (ODC) and arginine decarboxylase (ADC)^27^. Various other functions of polyamines have been proposed, including roles as secondary messengers of plant hormones^26^ and involvement in stress responses of plants and cyanobacteria^28–32^. The ability of biogenic polyamines to capture atmospheric CO_2_, reported by us for the first time, led us to examine the roles of biogenic polyamines in photosynthesis.

In this study, we investigated whether atmospheric CO_2_ captured by biogenic polyamines could be a substrate for the carboxylation reaction of Rubisco. If Rubisco could use CO_2_ incorporated in a polyamine solution as a substrate, then we could suggest an entirely new physiological function of polyamines. Therefore, we attempted to verify whether the carboxylation reaction occurs in the presence of a polyamine solution that has taken up CO_2_ from the atmosphere and that serves as a substrate for commercially available, partially purified Rubisco and crude Rubisco extracted from the terrestrial plant *Fallopia japonica*. Moreover, to verify how CO_2_ is incorporated from the atmosphere into the polyamine solution, we identified by nuclear magnetic resonance (NMR) the molecular species in the polyamine solution. We show that polyamines possibly contribute to CO_2_ diffusion and photosynthesis. Therefore, these findings should be useful both for elucidating novel physiological functions of polyamines and for developing new methods to reduce atmospheric CO_2_.

## Results

### Activation of Rubisco by polyamine solutions retaining CO_2_

**Figure 1:**
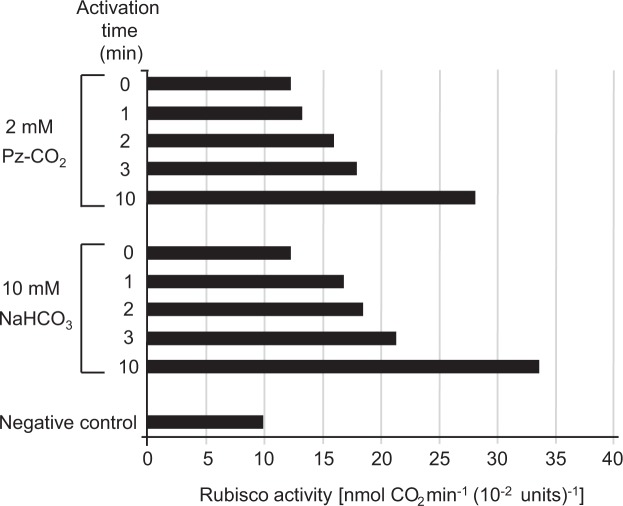
Comparison of Rubisco activation between piperazine (Pz) solutions retaining CO_2_ and a NaHCO_3_ solutions. Rubisco activity was measured following activation treatment with piperazine solutions retaining CO_2_ (Pz-CO_2_) or NaHCO_3_ solutions. The activation was carried out at 0 °C in the presence of 10 mM MgCl_2_ together with either 2 mM piperazine retaining CO_2_ or 10 mM NaHCO_3_ (see Methods). The bars represent Rubisco activity at each time point following the activation of an enzyme solution prepared using commercially available, partially purified Rubisco. The negative control denotes the data obtained for reactions in the absence of a CO_2_ supply excluding piperazine and NaHCO_3_ in both the activation buffer and reaction mixture.

Rubisco must be activated by CO_2_ and Mg^2+^ to function. Conditions consisting of 10 mM MgCl_2_, 10 mM NaHCO_3_, pH 7.8, and 0 °C for 10 min have been used for activation treatment^33^. To verify whether polyamines supply CO_2_ for Rubisco, we used polyamine solutions sufficiently incorporating CO_2_ for the activation treatment of Rubisco instead of NaHCO_3_ usually used as carbonate source. Figure 1 shows the comparison between Rubisco activation by piperazine and that by NaHCO_3_ using commercially available Rubisco. When activation was performed using CO_2_-incorporated piperazine, which is a non-natural cyclic diamine, Rubisco activity tended to increase proportionally to reaction time for 10 min. At 10 min after treatment, the activity was more than twice that at the beginning, and this time-dependent change was similar to that observed for the activation by NaHCO_3_. Therefore, the activation treatments were performed using piperazine at 0 °C for 10 min.

### Carboxylation reaction using CO_2_-incorporated polyamine solutions as substrates

**Figure 2:**
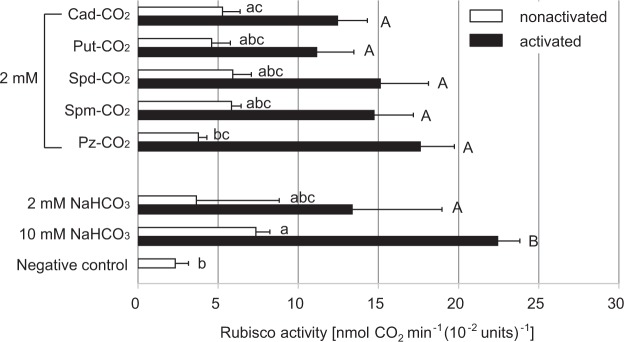
Polyamine solutions retaining CO_2_ act as substrates for Rubisco. The test polyamines were dissolved in water under atmospheric conditions and then stood at 20 °C for 2 days. The solutions incorporating atmospheric CO_2_ (Cad-CO_2_, Put-CO_2_, Spd-CO_2_, Spm-CO_2_ and Pz-CO_2_) were used as substrates for the carboxylation reaction of partially purified Rubisco. The open columns show Rubisco activity before the activation process (nonactivated), and closed columns indicate Rubisco activity after the activation process (activated). Lower-case and upper-case letters in the figure represent the results of statistical analyses of multiple comparisons using Fisher’s PLSD at the 5% significance level before and after the activation process. Bars indicate the standard error (10 mM NaHCO_3_: *n* = 6, 2 mM piperazine: *n* = 10, and other polyamines: *n* = 6). The lower part of the figure shows the Rubisco activity when NaHCO_3_ was used as a source of CO_2_ as well as when a CO_2_ source was not provided (negative control). The pH of the polyamine solutions retaining CO_2_ ranged from 8.9–9.1. Abbreviations used are: Cad, cadaverine: Put, putrescine: Spd, spermidine: Pz, piperazine.

To verify whether partially purified Rubisco can utilize CO_2_-incorporated polyamine solutions, we compared Rubisco activity that used different polyamines retaining CO_2_ instead of NaHCO_3_. The Rubisco activity was measured using a polyamine solution in which CO_2_ was taken up at 20 °C for 2 days to ensure sufficient equilibrium between the CO_2_ and polyamines. As shown in Fig. 2, compared with the NaHCO_3_ positive control treatment, the activation treatment markedly increased the activity of Rubisco for all CO_2_-incorporated polyamine solutions. The activity was 150–400% higher than that under the preactivation conditions. There were no significant differences in activity between CO_2_-incorporated polyamines and NaHCO_3_ [Fisher’s protected least significant difference (PLSD): *P* > 0.05] prior to the activation treatment, with the exception of piperazine. In addition, after the activation treatment, the Rubisco activity from all polyamines and NaHCO_3_ at the same concentrations was not significantly different (Fisher’s PLSD: *P* > 0.05). Thus, CO_2_-incorporated polyamine solutions were used as substrates for Rubisco.

### Influence of setting time on the incorporation of CO_2_ into polyamine solutions

Here, we investigated whether treatment with polyamine solutions at 20 °C for 2 days to facilitate the incorporation of CO_2_ into the solutions is appropriate. For this purpose, we compared differences in the activity of partially purified Rubisco at different setting times using piperazine solutions.

**Figure 3:**
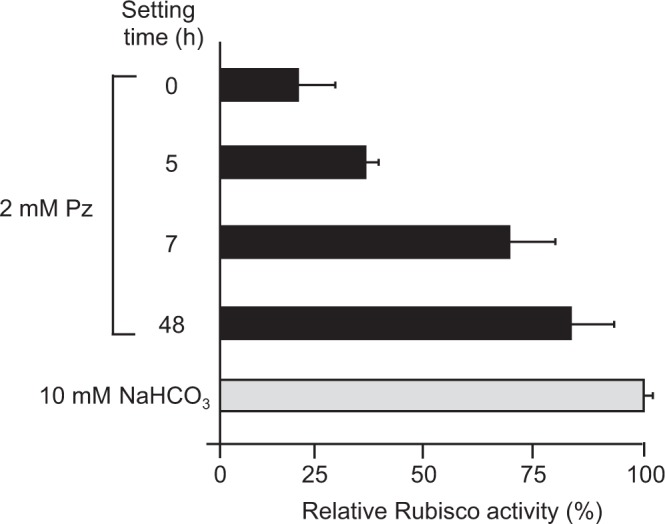
Influence of piperazine (Pz) solution setting time on Rubisco activity. Piperazine was dissolved in MilliQ water to a final concentration of 0.1 M and then stood at 20 °C for 48 hours to facilitate the uptake of atmospheric CO_2_ (pH ranged from 8.9–9.1). To measure its activity, partially purified Rubisco was pre-activated and used as part of an enzyme solution. Bars indicate the standard error (*n* = 3).

As shown in Fig. 3, the activity without pretreatment for incorporation was approximately 1/5 that when NaHCO_3_ was used as a substrate. The activity continued to increase from 5 to 7 hours; after 7 hours, the activity was approximately 3/4 that when NaHCO_3_ was used, which was not significantly different from that when piperazine was allowed to stand for 48 hours, as shown in Fig. 3 (t*-*test: *P* = 0.34).

### Carboxylation of crude Rubisco extracts

**Figure 4:**
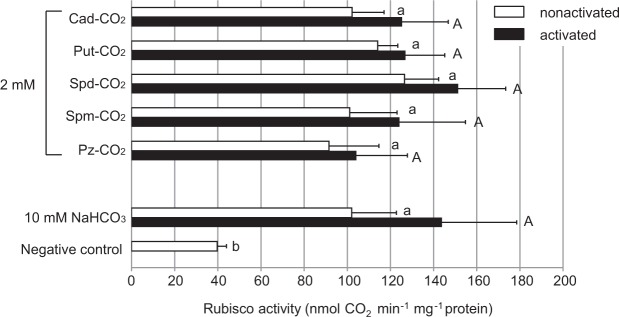
Activity of a crude extract of Rubisco prepared from the leaves of *F. japonica*. A solution containing test polyamines stood at 20 °C for 2 days to facilitate the uptake of atmospheric CO_2_ into the solution, after which the solution was used as a substrate for the crude enzyme extract prepared from the leaves of *F. japonica*. Refer to Fig. 2 for the measurement conditions and test methods for Rubisco activity (*n* = 4). Abbreviations used are: Cad, cadaverine: Put, putrescine: Spd, spermidine: Pz, piperazine.

Similar to our examination of partially purified Rubisco, we investigated whether Rubisco in a crude extract of the leaves of the terrestrial plant *F. japonica* could be used to examine a polyamine solution incorporating CO_2_ as a substrate. As shown in Fig. 4, no significant differences were observed between 10 mM NaHCO_3_ and any of the polyamine solutions tested (Fisher’s PLSD: *P* > 0.05). This trend was confirmed both before and after the activation process. In addition, following activation, the activity with polyamines as a substrate increased 10–20%, whereas that with NaHCO_3_ increased 40%.

### Relationship between Rubisco activity and concentrations of NaHCO_3_ as substrate

To investigate the relationship between the Rubisco activity and concentrations of NaHCO_3_, we compared the activity at 0 to 10 mM NaHCO_3_ used as substrates for the carboxylation reaction of partially purified Rubisco (Fig. S1). Rubisco activities without activation treatment were almost constant with NaHCO_3_ at more than 2 mM. On the other hand, Rubisco activities after activation treatment increased accompanying increasing concentration of NaHCO_3_.

### Effect of CO_2_-free polyamines on Rubisco activity

Solutions containing CO_2_-free polyamines were used as substrates together with NaHCO_3_ for the carboxylation reaction of partially purified Rubisco (Fig. S2). When the solutions containing polyamines and NaHCO_3_ each of 1 mM were used as substrates, the Rubisco activities were almost equal to that of 2 mM NaHCO_3_.

### Enhancement of 3-phosphoglycerate production by Rubisco activity assayed in putrescine-containing medium pre-bubbled with CO_2_ gas

**Figure 5:**
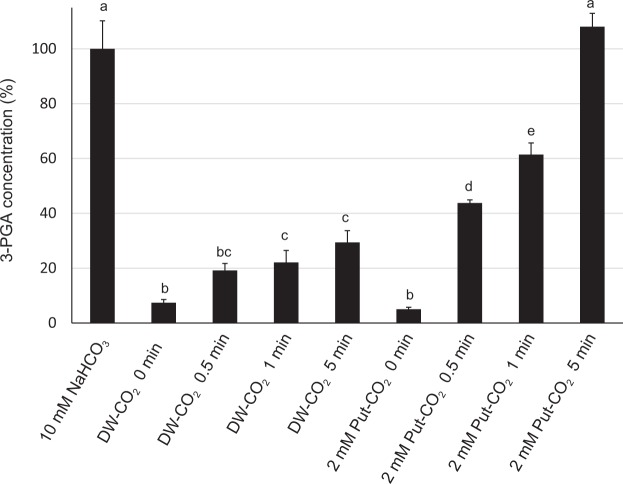
Enhancement of 3-phosphoglycerate production by Rubisco activity assayed in putrescine-containing medium pre-bubbled with CO_2_ gas. CO_2_ gas was passed through 10 mL either of 0.1 M putrescine (Put) solution or MilliQ water (DW) at room temperature for 0 to 5 min. The solutions incorporating CO_2_ were used as substrates for the carboxylation of partially purified Rubisco. To activate Rubisco, either 0.5 M (final 10 mM) NaHCO_3_, 0.1 M (final 2 mM) Put-CO_2_ (0, 0.5, 1 and 5 min), or DW-CO_2_ (0, 0.5, 1 and 5 min) was introduced to reaction solutions containing Rubisco. RuBP was added to each solution to initiate the reaction, then formic acid was added to each solution after 6 min to stop the reaction. Rubisco activity was analysed by LC-MS, detecting 3-PGA which is the direct carboxylation product of Rubisco. Different lower-case letters represent statistical significance at 5% in multiple comparisons using Tukey’s test. Bars indicate the standard errors (*n* = 3).

We examined whether or not the treatment with polyamine solutions passing through CO_2_ gas for 5 min was appropriately designed. CO_2_ gas (99.9%) was passed through 0.1 M putrescine solution and MilliQ water (DW) at 25 °C for 0, 0.5, 1 and 5 min, respectively. These solutions containing CO_2_, together with 10 mM NaHCO_3_ as the positive control, were introduced to partially purified Rubisco, and the mixtures were allowed to stand at room temperature for 10 min to activate Rubisco. RuBP was added to the mixtures to start the carboxylation reaction, then formic acid was added to stop the reaction after 6 min. 3-phosphoglycerate (3-PGA), a direct carboxylation product by Rubisco, was subsequently measured using LC–MS. When 10 mM NaHCO_3_ was used as a substrate, the concentration of 3-PGA in the resultant solution was 5.49 µg/L. Figure 5 shows the concentrations of 3-PGA after various periods in reaction as percentages of that with 10 mM NaHCO_3_. The DWs which had received CO_2_ gas produced 7, 19, 22 and 29% 3-PGA after 0, 0.5, 1, and 5 min, respectively. When the putrescine solution passing through CO_2_ gas at a final concentration of 2 mM was used as a substrate, the concentrations of 3-PGA were 5, 44, 61and 108% after 0, 0.5, 1, and 5 min, respectively. Thus, putrescine could capture CO_2_ quickly and efficiently provide CO_2_ as a substrate to Rubisco.

### Results of the NMR analysis of the uptake mechanisms of putrescine and piperazine

**Figure 6:**
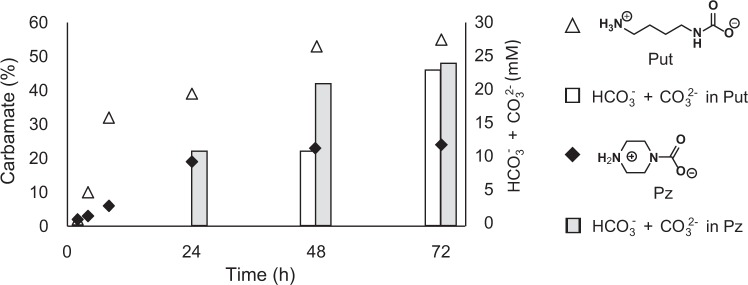
NMR analysis results of atmospheric CO_2_ incorporation by putrescine (Put) and piperazine (Pz) into aqueous solution. Changes in the relative amounts of the carbamate derivatives of amines (i.e., -CH_H2_NHCOO^−^) were determined following atmospheric CO_2_ uptake by putrescine and piperazine (50 mM/D_2_O), and changes in the amounts of HCO_3_^−^ + CO_3_^2−^ (bicarbonate and carbonate) in the aqueous solution were also determined. Carbamate derivatives and HCO_3_^−^ + CO_3_^2−^ were determined by ^1^H- and ^13^C-NMR spectra, respectively.

To analyze the mechanism of CO_2_ uptake by polyamines, 50 mM putrescine and piperazine were dissolved into deuterium oxide (D_2_O) and then allowed to stand at 20 °C for 2–72 hours. A fixed amount of 1,4-dioxane was added as an internal standard, and the concentrations of carbamate derivatives and bicarbonate plus carbonate (HCO_3_^−^ + CO_3_^2−^) were calculated from ^1^H-NMR and ^13^C-NMR spectra, respectively. As shown in Fig. 6, the proportion of carbamate derivatives increased beginning from the early stages. For putrescine, which is a primary diamine, the proportions of the carbamates were 2, 10, 32, 39, 53, and 55% at 2, 4, 8, 24, 48, and 72 hours, respectively. For piperazine, which is a secondary diamine, the proportions were 2, 3, 6, 19, 23, and 24% at 2, 4, 8, 24, 48, and 72 hours, respectively. In contrast, the HCO_3_^−^ and CO_3_^2−^ concentrations in the putrescine solution were detectable at 48 and 72 hours and were 11 and 23 mM, respectively. The HCO_3_^−^ + CO_3_^2−^ concentrations in the piperazine solution were detectable at 24, 48, and 72 hours and were 11, 21, and 23 mM, respectively.

### NMR analysis on the rate of CO_2_ uptake by putrescine in aqueous solution under 5% CO_2_ condition

We analyzes the rate of CO_2_ uptake by putrescine in aqueous solution under 5% CO_2_ condition. 50 mM putrescine in D_2_O were allowed to stand for 24 hours at 25 °C in a 5% CO_2_ incubator. As shown in Fig. S3, 41% of putrescine was changed to carbamate derivatives after 20 min. The carbamate derivatives increased until after 2 hours, reaching 20% after 24 hours due to the shift to cationic derivative and bicarbonate. These results indicate that putrescine had a rate of conversion to the carbamate derivative higher in 5% CO_2_ than atmospheric CO_2_.

## Discussion

Solutions containing synthetic amines, mainly alkanolamines, can capture CO_2_ at high concentrations in exhaust gases^34^. This method using artificial amines has been used since the 1930s in various industries to fix gaseous CO_2_ ^35^. In addition, another synthetic amine, polyethylene, can effectively capture CO_2_ from the air^36^. We recently showed that biogenic polyamines that are the most common biogenic amines can efficiently capture and dissolve atmospheric CO_2_ in aqueous solutions at 20 to 40 °C, as in the case of artificial amines^21^. Thus, we demonstrated that biogenic polyamines play important roles in the formation of diverse calcareous skeletons in marine organisms^21^. The ability of biogenic polyamines to capture atmospheric CO_2_ prompted us to examine the roles of biogenic polyamines in photosynthesis.

Our data clearly demonstrated for the first time that inorganic carbon either incorporated by polyamines from the air or derived from NaHCO_3_ can act as a substrate for partially purified Rubisco (Fig. 2). Polyamines incorporating CO_2_ could efficiently activate Rubisco and smoothly provide the CO_2_ as a substrate to Rubisco as shown in Figs 1 and 2. There results led us to conclude that polyamines retaining CO_2_ could smoothly supply this CO_2_ to the amino group in the active cite of Rubisco. The rate of the carboxylation reaction catalyzed by Rubisco increased with increasing setting time during which the polyamine solution took up CO_2_ from the air (Fig. 3). It was also found that the substrate of this reaction is derived from atmospheric CO_2_, as the carboxylation reaction did not occur by piperazine alone (Fig. 3). This result also showed that the activation and carboxylation reaction of Rubisco is due to CO_2_ supply from polyamine solution containing carbamate derivatives and carbonates, but not due to the increase of pH in the reaction solution. High Rubisco activity similar to that recorded when partially purified Rubisco was used was observed even when crude extracts of *F. japonica* were used as enzyme solutions (Fig. 4). Regardless of which polyamine-CO_2_ solution was used as a substrate, the activity obtained did not significantly differ from that observed when NaHCO_3_ was used. As shown in the lower part of Fig. 4, instances occurred in which moderate activity was detected even when an inorganic carbon source was not provided. This phenomenon probably occurred because the tissues of the organism contained a relatively large amount of biogenic polyamines. Also, atmospheric CO_2_ is easily dissolved in the crude extract solution, even though this activity is marginal.

Figures 1 to 4 show Rubisco activities measured by the decrease in absorbance at 340 nm for NADH, accompanying the reduction of 1,3-bisphosphoglycerate (1,3-BPGA), which is a phosphorylation product of 3-PGA. Figure 5 further shows the concentrations of 3-PGA, the direct product of the carboxylation reaction of Rubisco, measured by LC-MS. Polyamines react with CO_2_ in aqueous solutions, forming carbamates. The carbamate bond thus formed, though covalent, is reversible and easily releases CO_2_ ^37^. Due to such characteristics of carbamate, putrescine solution quickly captured CO_2_ gas within at least 5 min, and efficiently provided the incorporated CO_2_ to Rubisco as shown in Fig. 5.

The polyamines tested in this study contain different numbers of amino groups: spermine has four amino groups in one molecule, spermidine has three, and putrescine and cadaverine both have two. Piperazine is a non-natural cyclic diamine and was used for comparison. Polyamines having a larger number of amino groups incorporate more CO_2_; however, in the experiments using partially purified and crude Rubisco extracts, no statistically significant differences among these polyamines were detected (Figs 2 and 4). Furthermore, polyamines were found to have ability of receiving CO_2_ from NaHCO_3_ as well as atmospheric CO_2_. Although 2 mM NaHCO_3_ was not a substrate concentration that sufficiently saturates the carboxylation rate of Rubisco as shown in Fig. S1, the Rubisco activities were equal to or higher than those at 2 mM NaHCO_3_, when 1 mM NaHCO_3_ plus 1 mM of polyamines were used as substrates (Fig. S2). The NMR analysis results showed that the primary diamine putrescine had a faster rate of conversion to the carbamate derivative than did the secondary diamine piperazine; after 48 hours, more than 50% of primary diamine had been converted to the carbamate derivative where the putrescine solution contained 10 mM carbonate species (Fig. 6). This phenomenon is presumably because the formation of carbamate derivatives is more likely to occur for a primary amine and because the resulting carbamate derivative is more stable. The carbamate derivative gradually shifts to the more stable HCO_3_^−^. Approximately 20% of piperazine, a non-natural secondary diamine, was converted to the carbamate derivative after 72 hours, and the rate of HCO_3_^−^ production was faster than that for putrescine. This phenomenon is probably because the carbamate derivative of the secondary amine is less stable. These results indicated that the carbamate derivatives of primary diamine biogenic polyamines may be relatively stable and more easily release CO_2_ than dose bicarbonate ion only because of difference in their stability. Polyamines shown in Fig. 6 were reacted with atmospheric CO_2_ in a 48-well plate with a cover, thus, the carbamate formation rate was slow because of restricted air. We reported previously that polyamines could actively capture atmospheric CO_2_ and facilitate a bacterial extracellular CaCO_3_ precipitation^21^. When marine bacteria were cultured on the petri dish under airtight conditions, CaCO_3_ precipitation was not observed. However, once the petri dish was exposed to air, CaCO_3_ was smoothly formed on the agar broth within a day. Based on these results, we concluded that polyamines facilitated the incorporation of CO_2_ from the air into the culture medium^21^. We consider that the formation rate of carbamate derivatives of polyamines have the same order as Rubisco activation, because the reaction mechanisms of CO_2_ absorption by polyamines are the same as that of the activation of Rubisco. Actually, when polyamines were reacted with 5% CO_2_, the rate of carbamate formation had minute order as shown in Fig. S3. There are many kinetic studies for the formation rate of carbamate derivative in synthetic amines^34,38^, which are consistent with our results. Moreover, we roughly calculated the concentration of dissolved CO_2_ in Rubisco reaction solutions. It has been reported that 0.1 M bicine (pH 8.0) containing 5 mM MgCl_2_ has p*K*_1_ = 6.22^39^. Therefore, the concentration of dissolved CO_2_ in the 10 mM NaHCO_3_ solution which was used as a positive control in the present study is roughly calculated to be 105 µM by the Henderson-Hasselbalch equation. As shown in Fig. 6, the CO_2_-saturated polyamine solution contained 50% each of [HCO_3_^−^ + CO_3_^2−^] and carbamate derivatives. Therefore, the CO_2_-saturated solution in 2 mM polyamine used in the Rubisco assays is supposed to contain 1 mM HCO_3_^−^ and dissolved CO_2_ at 10.5 µM. Thus, the amount of CO_2_ generated from 1 mM HCO_3_^−^ is quite low compared with that generated from 10 mM NaHCO_3_ which was used as the positive solution. Nevertheless, the Rubisco activity in the CO_2_-saturated solution in 2 mM polyamines was almost the same to or even higher than that in 10 mM NaHCO_3_ (Fig. 5). Thus, the most part of dissolved CO_2_ is considered to be generated from the carbamate derivatives of polyamines.

In addition, during the activation of Rubisco, the polyamine solutions retaining CO_2_ showed the same effects as did NaHCO_3_ solutions, which are normally used as CO_2_ sources (Figs 1, 2 and 4). Rubisco is activated under weakly alkaline conditions^40–46^ and occurs via the conversion of the amino group of a lysine residue at position 201 to a carbamate derivative; it is likely that this reaction occurs only under alkaline conditions. The polyamine solution that absorbed CO_2_ is weakly alkaline at pH 8.5–9.1, and many carbamate derivatives and HCO_3_^−^ molecules exist in the solution. Therefore, it is likely that Rubisco is strongly activated, and carbonate species may be provided to serve as a substrate. However, there are several papers available, reporting that the substrate of Rubisco is CO_2_, but not HCO_3_^−^. For example, Cooper *et al*.^47^ demonstrated that CO_2_ is a better substrate for Rubisco than HCO_3_^−^. In the present study, we used NaHCO_3_ as a carbon souse for positive control in *in vitro* Rubisco assays, but not CO_2_ gas, because we aimed to know the exact concentration of dissolved inorganic carbons. Under the present weakly alkaline conditions, the carboxylation reaction of Rubisco proceeded smoothly in the presence of NaHCO_3_ at an mM order. This fact suggests that the sufficient amount of CO_2_ was produced by the equilibrium reaction from NaHCO_3_ and used as a substrate in the CO_2_ fixation reaction by Rubisco. In the polyamine solution retaining carbamate and bicarbonate ion, CO_2_ will be generated by the equilibrium reaction from these carbamate and bicarbonate ion. Okabe *et al*.^40^ reported that hydroxylamine also enhances Rubisco activity with similar alkalinisation mechanisms that we note in the presence study at CO_2_-free polyamines experiments as shown in Fig. S2. However, this substance is highly toxic and thus difficult to exist at high concentrations in intact cells^48^. In contrast, polyamines are biogenic amines and exist at high concentrations within the cells. Furthermore, polyamines can directly capture atmospheric CO_2_ in aqueous solution. Thus, our findings about polyamines that can play functional roles in photosynthesis are quite novel.

Our data suggest that polyamines might facilitate inorganic carbon transport from cell surfaces to Rubisco, as polyamines ensure high concentrations of retained inorganic carbon species in leaf cells. Therefore, CO_2_ diffusion in leaves is the rate-limiting factor for photosynthesis in leaves^5–9^, and it is conceivable that polyamines could contribute to photosynthesis by retaining inorganic carbon species. Furthermore, if the polyamine concentration in leaves could be increased, CO_2_ could be efficiently taken up even when stomata are slightly open; this phenomenon may help prevent moisture loss from the stomata during desiccation and suggests that polyamines may also be involved in drought tolerance of plants^32^. As mentioned before, chloroplasts contain a large amount of polyamines with ODC and ADC, both of which are main polyamine biosynthetic enzymes^26,27^. Based on our results, we speculate the role of polyamines in inorganic carbons concentration mechanisms in plants as follows. The high concentrations of polyamines in the cytosol and chloroplasts provide high concentration of intracellular inorganic carbons, where CO_2_ generated by the equilibrium reaction from these inorganic carbons (carbamates) and bicarbonate ion will be used as a substrate of Rubisco. Furthermore, a part of the polyamines synthesized in the cytosol permeate the intercellular air spaces through polyamine transporters^49^. These polyamines could capture CO_2_ contained in intercellular air spaces and transport to intercellular fluid, then contribute to CO_2_ diffusion. Of course, a large amount of CO_2_ is well known to be produced by carbonic anhydrase from bicarbonate and used as a substrate of Rubisco *in vivo*.

Moreover, the ability of polyamines to produce bicarbonate-carbonate in aqueous solution decreases at low temperatures because this ability requires high temperatures for the efficient hydration reaction of the carbamate derivatives to bicarbonate^21^. This property of polyamines might be related to the promotion of polyamine biosynthesis during exposure to low temperatures^28^. This novel mechanism for CO_2_ fixation by Rubisco involving biogenic polyamines provides a new strategy for photosynthetic research and suggests a new CO_2_-removal concept that could reduce both atmospheric CO_2_ levels and global warming.

## Methods

### Preparation of polyamine solutions for Rubisco assay

To incorporate atmospheric CO_2_ into polyamine solutions, solutions (2 ml) containing piperazine (Wako Pure Chemicals, Osaka, Japan) and biogenic polyamines (putrescine, spermidine, cadaverine and spermine; Wako Pure Chemicals, Osaka, Japan) each at 0.1 M were added to multidishes (24 wells, diameter of 15 mm), which stood for 48 hours at 20 °C. The resultant polyamine solutions at pH 8.9–9.1 were used as carbonate sources in Rubisco assays.

To activate Rubisco, the enzyme was preincubated in the presence of the polyamine solutions retaining CO_2_ instead of NaHCO_3_ solutions^33^.

To investigate the time-dependent changes in Rubisco activity, solutions (2 ml) containing 0.1 M piperazine were added to multidishes (24 wells, diameter of 15 mm), which stood for 0, 5, 7 and 48 hours at 20 °C. The resultant piperazine solutions at pH 8.9–9.1 were used as carbonate sources in Rubisco assays. Ten millimolar solutions of NaHCO_3_ served as positive controls.

### Rubisco assays

Partially purified Rubisco (0.05 units mg^−1^ solid; Sigma-Aldrich, St. Louis, MO, USA) and crude extracts from the leaves of *F. japonica* Houtt. var. japonica were used in Rubisco assays.

The partially purified Rubisco was dissolved in buffer [100 mM hydroxyethyl piperazineethanesulfonic acid (HEPES), 10 mM dithiothreitol (DTT), 5 mM MgCl_2_ and 1 mM EDTA, pH 7.8] at 10 m units 75 µl^−1^ as an enzyme solution. The buffer was equilibrated with pure N_2_ gas in order to exclude CO_2_.

The leaves of *F. japonica* were collected on 25 September 2012 at Kitasato University (Sagamihara, Kanagawa, Japan). Two leaf discs (0.49 cm^2^ each) were punched, frozen immediately in liquid N_2_, and then maintained at −80 °C until assays. Two frozen leaf discs were rapidly homogenized in a chilled mortar filled with 1 ml of CO_2_-free extraction buffer [100 mM HEPES, 10 mM DTT, 5 mM MgCl_2_, 1 mM EDTA, 2% (w/v) PVP40, 1% (v/v) Triton X-100 and 0.2 mM leupeptin, pH 7.8] for 3 min. The extraction buffer was equilibrated with pure N_2_ gas in order to exclude CO_2_ prior to the extraction. The homogenate was centrifuged at 17400 × *g* for 2 min at 4 °C, after which the supernatant was used immediately to assay the initial activity of Rubisco (nonactivated).

The total activity of Rubisco (activated) was also determined following the activation of a 160 µl enzyme solution and was achieved by preincubation for more than 10 min at 0 °C in 40 µl of the activation buffer (75 mM HEPES, 10 mM MgCl_2_ and 10 mM NaHCO_3_ or 2 mM polyamine solution).

Rubisco activity was determined in accordance with the spectrophotometric method of Lilley and Walker^50^, which was partly modified by Sakata *et al*.^51^. The reaction mixture (100 mM bicine buffer containing 5 mM MgCl_2_, 5 mM creatine phosphate, 1 mM ATP, 5 units ml^−1^ creatine kinase, 5 units ml^−1^ 3-phosphoglycerate kinase, 5 units of glyceraldehyde-3-phosphate dehydrogenase and 0.1 mM NADH, pH 8.2) was treated with pure N_2_ gas for 30 min in order to exclude CO_2_. All chemicals and enzymes were commercially available. A 2925 µl reaction mixture that contained 150 µl of 6 mM RuBP and either 60 µl of 0.5 M NaHCO_3_ or 0.1 M polyamine solutions was added to a cuvette under a N_2_ gas atmosphere at 25 °C. After confirming no more decease in the absorbance at 340 nm, the Rubisco activity was measured by the addition of 75 µl of partially purified Rubisco or the crude extract to the cuvette. Approximately 5 min elapsed between the start of homogenization and the start of assay. Rubisco activity was recorded by the decrease in absorbance at 340 nm and was corrected by a blank assay, in which the reaction mixture did not contain RuBP. To determine Rubisco activity per unit protein, the protein content in the extract was assayed according to the method of Bradford^52^ using a protein assay kit (BIO-RAD, Hercules, CA, USA).

### Rubisco activity in putrescine solution passing through CO_2_ gas as detected with the increased concentrations of 3-PGA by LC-MS

CO_2_ gas (99.9%) was passed through 10 mL either of 0.1 M putrescine solution or MilliQ water, which had been pre-treated with N_2_ gas to exclude CO_2_, for 0, 0.5, 1 and 5 min at room temperature. The solutions incorporating CO_2_ were used as substrates for the carboxylation reaction of partially purified Rubisco. A 905 µl of CO_2_-free reaction mixture (0.1 M bicine, pH 8.2, containing 5 mM MgCl_2_), 25 µl of the enzyme solution of Rubisco, and either 20 µl of 0.5 M NaHCO_3_, 0.1 M putrescine solutions incorporating CO_2_, or MilliQ water were introduced to 1.5 mL tube. The mixed solutions were allowed to stand for 10 min at 25 °C to activate Rubisco. Then, each of the solutions were added with 50 µl of 6 mM RuBP to start the reaction, and after 6 min later with 200 µl of formic acid to stop the reaction. 3-phosphogriseric acid (3-PGA), the direct product of the carboxylation reaction of Rubisco was measured using LC–MS. In brief, high-resolution hybrid quadrupole-time-of-flight mass spectrometer (Triple TOF 5600+, SCIEX) operated in a negative ion mode was coupled to reversed phase chromatography via electrospray ionization and scanned from *m*/*z* 50 to 600 at high resolution. LC separation was achieved on a InertSustain C18 column (2.1 mm × 150 mm, 3 μm particle size, GL Sciences) using a gradient of solvent A (10 mM tributylamine + 10 mM acetic acid in water) and solvent B (methanol). The gradient was: 0 min, 0% B; 1 min, 0% B; 1.5 min, 15% B; 3 min, 15% B; 8 min, 50% B; 10 min, 100% B; 11 min, 100% B; 11.5 min, 0% B; 17 min, 0% B at a flow rate of 200 μl min^−1^. Quantification of 3-PGA for its deprotonated molecule [M–H^+^]^−^ at *m/z* 184.9857 was performed using MutliQuant integration software (SCIEX). The standard compound of 3-PGA was purchased from Sigma-Aldrich (USA).

### NMR analysis of the uptake mechanisms of putrescine and piperazine

Two-milliliter solutions of D_2_O containing 50 mM putrescine or 50 mM piperazine were added to multidishes (24 wells, diameter of 10 mm). The multidishes stood at 20 °C for 2, 4, 8, 24, 48, or 72 hours. The resultant carbamate and HCO_3_^−^ + CO_3_^2−^ (bicarbonate and carbonate) were characterized by using ^1^H-NMR and ^13^C-NMR^21,53–55^. An internal standard of 1,4-dioxane (5 µl, diluted 10-fold with D_2_O) was added to each NMR sample. The yield of carbamate derivatives was estimated by the ^1^H-NMR spectrum based on the area ratio of integration beneath the peaks for the α-methylene proton of the amines and their carbamates^21,53^. To determine the concentrations of HCO_3_^−^ + CO_3_^2−^, solutions of D_2_O containing 5 to 50 mM NaHCO_3_ were measured with ^13^C-NMR^54,55^. A calibration curve was then obtained from the area ratio of integration beneath the peaks for both dioxane and HCO_3_^−^ + CO_3_^2−^ from the spectra of the NaHCO_3_ solutions, and the concentration was estimated by the ratio of dioxane to HCO_3_^−^ + CO_3_^2−^ in each sample. The ^1^H- and ^13^C-NMR spectra were recorded using a Bruker AVANCE (600 and 700 MHz) spectrometer.

### NMR analysis on CO_2_ incorporation by putrescine in aqueous solution under 5% CO_2_ condition

To examine the reactivity of putrescine under 5% CO_2_ condition, one-milliliter solutions of D_2_O containing 50 mM putrescine were introduced into multidishes (24 wells, diameter of 10 mm). The multidishes stood at 25 °C for 24 hours in a 5% CO_2_ incubator. The resultant carbamates were characterized by using ^1^H-NMR as shown in Fig. S3.

## Electronic supplementary material

Supplementary Figure S1-3
