# Supplementary material for: Atmospheric CO2 captured by biogenic polyamines is transferred as a possible substrate to Rubisco for the carboxylation reaction
Source: Sci Rep. 2018 Dec 7;8:17724. doi: 10.1038/s41598-018-35641-8 (PMC6286370; doi:10.1038/s41598-018-35641-8)
Supplement: Supplementary file 1 — Supplementary Figure S1-3 [file 41598_2018_35641_MOESM1_ESM.doc]

**Supplementary Figure S1-3**

**Title:** Atmospheric CO_2_ captured by biogenic polyamines is transferred as a possible translated substrate to Rubisco for the carboxylation reaction

**Journal:** Scientific Reports

**Authors:** *****Ko Yasumoto^1^, *****Tsuyoshi Sakata^2^, Jun Yasumoto^3^, Mina Yasumoto-Hirose^4^, Shun-ichi Sato^1^, Kanami Mori-Yasumoto^5^, Mitsuru Jimbo^1^, Takenori Kusumi^6^, Shugo Watabe^1^

**Corresponding authors (*):**

Ko Yasumoto, Kitasato University School of Marine Biosciences, 1-15-1 Kitasato, Minami, Sagamihara, Kanagawa 252-0373, Japan.

E-mail: yasumoto@kitasato-u.ac.jp

Tsuyoshi Sakata, Biological Laboratory, Center for Natural Sciences, College of Liberal Arts and Sciences, Kitasato University, 1-15-1 Kitasato, Minami, Sagamihara, Kanagawa 252-0373, Japan.

E-mail: sakata@kitasato-u.ac.jp

**Supplementary Figure S1. Relationship between Rubisco activity and concentrations of NaHCO_3_ as substrate.** NaHCO_3_ solutions of 0 to 10 mM were used as substrates for the carboxylation reaction of partially purified Rubisco. Open squares show Rubisco activity before the activation process (nonactivated), and closed squares indicate that after the activation process (activated).

**Supplementary Figure S2.** **Effect of CO_2_-free polyamines on Rubisco activity.** Solutions containing 1 mM polyamines incorporating no CO_2_ (Cad, Put, Spd, Spm or Pz at pH 8.9～9.1) and 1 mM NaHCO_3_ were used as substrates for the carboxylation reaction of partially purified Rubisco. Open columns show Rubisco activity before the activation process (nonactivated), and closed columns indicate that after the activation process (activated).

**Supplementary Figure S3. NMR analysis on CO_2_ incorporation by putrescine in aqueous solution under 5% CO_2_ condition.** One-milliliter solutions of D_2_O containing 50 mM putrescine were added to multidishes (24 wells, diameter of 10 mm). The multidishes stood at 25 °C for 24 hours in a 5% CO_2_ incubator. Changes in the relative amounts of the carbamate derivatives of putrescine were determined following CO_2_ uptake by putrescine (50 mM/D_2_O) in the aqueous solution. The carbamate yield was estimated by ^1^H-NMR measurement based on an area ratio of integration beneath peaks for the α methylene (i.e., CH_2_N) proton of putrescine and its carbamate.
